# Activity and safety of SHR3680, a novel antiandrogen, in patients with metastatic castration-resistant prostate cancer: a phase I/II trial

**DOI:** 10.1186/s12916-022-02263-x

**Published:** 2022-03-04

**Authors:** Xiaojian Qin, Dongmei Ji, Weijie Gu, Weiqing Han, Hong Luo, Chuanjun Du, Qing Zou, Zhongquan Sun, Chaohong He, Shaoxing Zhu, Tie Chong, Xin Yao, Ben Wan, Xinfeng Yang, Aobing Bai, Chunlei Jin, Jianjun Zou, Dingwei Ye

**Affiliations:** 1 Department of Urology, Fudan University Shanghai Cancer Center, 270 Dong’an Road, Shanghai, 200032 China; 2 Department of Medical Oncology, Fudan University Shanghai Cancer Center, Shanghai, China; 3 Department of Urology, The Affiliated Cancer Hospital of Xiangya School of Medicine, Central South University, Hunan Cancer Center, Changsha, China; 4 Department of Urology, Chongqing University Cancer Hospital, Chongqing Cancer Hospital, Chongqing, China; 5 Department of Urology, The Second Affiliated Hospital of Zhejiang University School of Medicine, Hangzhou, China; 6 Department of Urology, Jiangsu Cancer Hospital, Nanjing, China; 7 Department of Urology, Huadong Hospital Affiliated to Fudan University, Shanghai, China; 8 Department of Urology, Affiliated Cancer Hospital of Zhengzhou University, Zhengzhou, China; 9 Department of Urology, Zhejiang Cancer Hospital, Hangzhou, China; 10 Department of Urology, The Second Affiliated Hospital of Xi’an Jiaotong University, Xi’an, China; 11 Department of Urology, Tianjin Medical University Cancer Institute & Hospital, Tianjin, China; 12 Departmeint of Urology, Beijing Hospital, Beijing, China; 13 Department of Clinical Development, Jiangsu Hengrui Pharmaceuticals Co., Ltd., Shanghai, China

**Keywords:** Androgen-receptor antagonist, Castration-resistant prostate cancer, SHR3680, Phase 1/2 study, Prostate-specific antigen response

## Abstract

**Background:**

Antagonizing the androgen-receptor (AR) pathway is an effective treatment strategy for patients with metastatic castration-resistant prostate cancer (CRPC). Here, we report the results of a first-in-human phase 1/2 study which assessed the safety, pharmacokinetics, and activity of SHR3680 (a novel AR antagonist) in patients with metastatic CRPC.

**Methods:**

This phase 1/2 study enrolled patients with progressive metastatic CRPC who had not been previously treated with novel AR-targeted agents. In the phase 1 dose-escalation portion, patients received oral SHR3680 at a starting daily dose of 40 mg, which was subsequently escalated to 80 mg, 160 mg, 240 mg, 360 mg, and 480 mg per day. In phase 2 dose-expansion portion, patients were randomized to receive daily dose of 80 mg, 160 mg, or 240 mg of SHR3680. The primary endpoint in phase 1 was safety and tolerability and in phase 2 was the proportion of patients with a prostate-specific antigen (PSA) response (≥ 50% decrease of PSA level) at week 12.

**Results:**

A total of 197 eligible patients were enrolled and received SHR3680 treatment, including 18 patients in phase 1 and 179 patients in phase 2. No dose-limiting toxicities were reported and the maximum tolerated dose was not reached. Treatment-related adverse events (TRAEs) occurred in 116 (58.9%) patients, with the most common one being proteinuria (13.7%). TRAEs of grade ≥ 3 occurred in only 23 (11.7%) patients, and no treatment-related deaths occurred. Antitumor activities were evident at all doses, including PSA response at week 12 in 134 (68.0%; 95% CI, 61.0–74.5) patients, stabilized bone disease at week 12 in 174 (88.3%; 95% CI, 87.2–95.5) patients, and responses in soft tissue lesions in 21 (34.4%, 95% CI, 22.7–47.7) of 61 patients.

**Conclusion:**

SHR3680 was well tolerated and safe, with promising anti-tumor activity across all doses tested in patients with metastatic CRPC. The dose of 240 mg daily was recommended for further phase 3 study.

**Trial registration:**

Clinicaltrials.gov NCT02691975; registered February 25, 2016.

**Supplementary Information:**

The online version contains supplementary material available at 10.1186/s12916-022-02263-x.

## Background

Prostate cancer ranks the second most commonly occurred malignancy and the fifth leading cause of cancer-related death in men, accounting for 14.1% of total newly diagnosed cancer and 6.8% of total cancer death in men worldwide [1]. The growth of prostate cancer cells is androgen-dependent and castration therapy is used as the initial treatment for patients with advanced prostate cancer [2]. However, nearly all castrated patients will inevitably develop castration-resistant prostate cancer (CRPC), mainly due to the persistent activation of androgen receptor (AR) signaling pathway [3–5].

The approvals of two second-generation of AR antagonists enzalutamide and apalutamide in the treatment of CRPC by FDA have greatly revolutionized the treatment paradigm of this disease [6–9]. Despite of promising benefits, some patients do not respond to the approved drugs or only have limited response duration. On the other hand, the risk of seizure is considered as a main safety issue of the second-generation of AR antagonists, which may be attributed to the off-target inhibition of γ-aminobutyric acid (GABAa) receptor by drugs penetrated through the blood-brain barrier [10, 11].

SHR3680, a novel AR antagonist, preclinically displayed comparable anti-tumor potency but with much less distribution in the brain and significantly decreased risk to induce seizure compared with enzalutamide. Based on this context, we conducted this first-in-human phase 1/2 study to assess the safety, pharmacokinetics (PK), and activity of SHR3680 in patients with metastatic CRPC.

## Methods

### Study design and participants

This was a multicenter trial with a phase 1 dose-escalation portion and a phase 2 dose-expansion portion, which recruited patients at 11 hospitals in China (Additional file 1: Table S1). This trial is registered with ClinicalTrials.gov, number NCT02691975.

Eligible patients were men aged 18–80 years, had histological diagnosis of prostatic adenocarcinoma, progressed on (or were intolerant to or unwilling to receive) previous docetaxel-containing chemotherapy, had a castrate level of testosterone ≤ 50 ng/dL or 1.73 nmol/L, had an Eastern Cooperative Oncology Group (ECOG) performance status of 0 or 1, had a life expectancy of at least 6 months, and had adequate organ function. Disease progression was defined as meeting at least one of prostate-specific antigen (PSA) progression (≥ 3 rising PSA levels with an interval of ≥ 1 week and the last results of ≥ 2 ng/mL), soft tissue progression according to Response Evaluation Criteria in Solid Tumors (RECIST) guidelines v1.1, or bone disease progression according to Prostate Cancer Working Group (PCWG2) criteria despite androgen-deprivation therapy. Key exclusion criteria included prior treatment with second-generation of AR antagonists, abiraterone acetate, or ketoconazole; prior history of seizure or diseases that predispose to seizure.

### Procedures

In the phase 1 dose-escalation portion, patients were sequentially assigned to different doses to determine the maximum tolerable dose (MTD) in a 3+3 design. Patients received oral SHR3680 at a starting daily dose of 40 mg, which was subsequently escalated to 80 mg, 160 mg, 240 mg, 360 mg, and 480 mg per day. Each cycle contained 28 days of continuous administration. Three or six patients were included in each dose group. If dose-limiting toxicity (DLT) occurred in one of the first three patients in a certain dose group, three more patients would be further enrolled; if one more patient had DLT, escalation to higher dose was terminated, and MTD was defined as the previous dose.

The phase 2 portion contained two stages of dose-expansion. In the first stage, patients were randomized to receive 80 mg, 160 mg, and 240 mg per day of SHR3680, with 33–36 patients in each dose group. Afterwards, 160 mg and 240 mg per day were further selected for use in the second stage of dose-expansion, and 35–45 additional patients were randomized to each group. Randomization was conducted using clinical trial randomized grouping system of Nanjing Medical University. The doses for expansion were selected on the basis of tolerability, safety, and PK of each dose by investigator and funder. Randomization were stratified by prior chemotherapy (yes vs no) and number of bone metastases (≤ 5 vs > 5).

Treatment was continued until radiographic progression, unacceptable toxicity, investigator decision, or withdrawal of consent, whichever occurred first. For patients who experienced a grade ≥ 3 hematological adverse event (AE) or a grade ≥ 2 nonhematological adverse event toxicity that is attributed to the study drug, treatment could be interrupted. When the toxicity recovered to grade ≤ 1, treatment could be resumed at the original dose (40 mg per day) or a lower dose. For patients with treatment interruption > 14 days, the treatment must be discontinued.

### Assessment

PSA measurements were performed on days 15 and 28 of cycle 1, on days 28 of cycle 2–6, once every 3 cycles thereafter. PSA progression should be confirmed by a second PSA level measurement at least 3 weeks later. Soft tissue response was evaluated according to RECIST v1.1 using CT or MRI, and the first complete response (CR) or partial response (PR) required confirmation 4 weeks later. Radiological assessments were conducted every 3 treatment cycles. Bone disease was assessed according to PCWG2 criteria using radionuclide bone scan. AEs were graded based on the Common Terminology Criteria for Adverse Events, v4.03. After discontinuation of treatment, patients were followed up every 3 months to assess survival and AE assessment were further performed within 30 days after the last administration.

A total of 42 patients were involved in PK analysis, including 18 patients from dose-escalation portion (3 patients in each dose group) and 24 patients from dose-expansion portion (80 mg, 160 mg, and 240 mg per day; 8 patients in each group). A single dose of SHR3680 was firstly given on day 1 and continuous daily dosing was started from day 8. Blood samples were collected at pre-dose, after the single dose, and during the continuous administration period of SHR3680 for PK analysis. Samples were shipped frozen, and the SHR3680 concentration was analyzed using liquid chromatography-tandem mass spectrometry.

### Outcomes

The primary endpoints were DLT, MTD, and proportion of patients with a PSA response (defined as a decrease of ≥ 50% in PSA from baseline) at week 12. The secondary endpoints included proportion of patients with a PSA response during the treatment, the best changes of PSA level from baseline, time to PSA progression, objective response rate (ORR, proportion of patients whose best overall response was complete or partial response according to RECIST v1.1), disease control rate (DCR, proportion of patients whose best overall response was complete response, partial response, or stable disease according to RECIST v1.1), radiological progression-free survival (PFS, defined as time from first dose until soft-tissue disease progression [RECIST v1.1], bone lesion progression [PCWG2 criteria], or death, whichever occurred first), overall survival (OS, the time from first dose to death from any cause), time to first subsequent therapy (TFST), proportion of patients with stable disease in bone at week 12, safety, and PK.

### Statistical analysis

No hypothesis test for this study. For dose-escalation part (phase 1), the sample size was determined according to the dose-escalation rules of 3+3. For dose expansion part (phase 2), the sample size for each dosage (80 mg, 160 mg, and 240 mg per day) was 33–36 at stage 1 and additional 35–45 patients added for each 160 mg and 240 mg per day dosage at stage 2. Efficacy (except ORR and DCR) and safety were assessed in a population which consisted of all patients who received at least one dose of SHR3680 treatment. ORR and DCR were assessed based on the population who had measurable disease at baseline. PK was analyzed in patients with sufficient number of data points for determining drug concentration or PK parameters.

PSA response rate at week 12, proportion of patients with stable disease in bone at week 12, ORR, and DCR were presented with their corresponding 95% CIs calculated using Clopper-Pearson method. Median time to PSA progression, TFST, PFS, and OS were estimated with the Kaplan-Meier method, and their accompanying 95% CIs were calculated using Brookmeyer-Crowley method. Statistical analyses of efficacy and safety were performed using the SAS software (v9.4), and PK analysis for all parameters was performed using Phoenix WinNonlin (v8.0 or higher).

## Results

### Patients

**Fig. 1:**
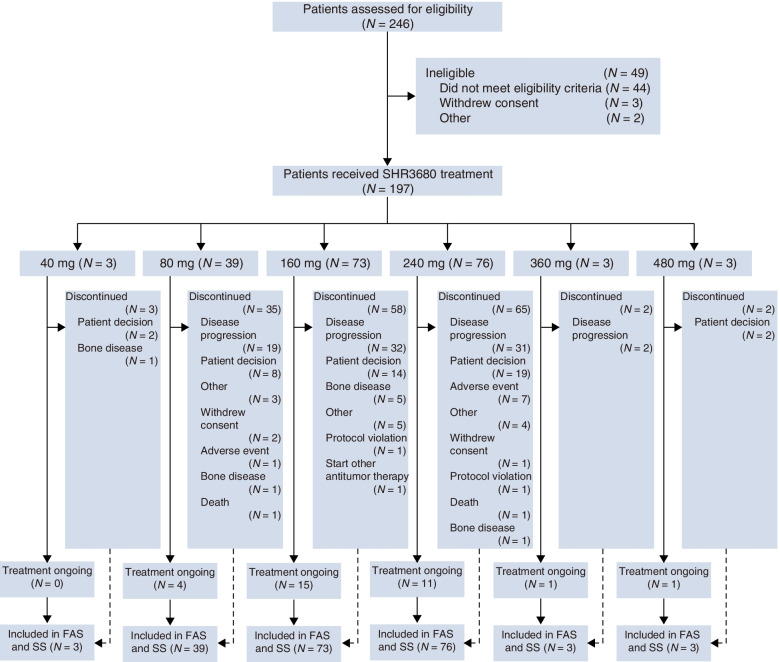
Study profile

Between March 28, 2016, to October 5, 2018, 246 patients were screened for eligibility and 197 patients were enrolled, including 18 patients (3 patients in each dose group) in dose-escalation portion and 179 patients in dose-expansion portion. All the 197 patients received at least one dose of SHR3680 treatment and were therefore included in full analysis population and safety analysis population (Fig. 1).

As of data cutoff on March 01, 2020, with a median follow-up of 19.2 months (range, 1.3–43.7), the duration of exposure to SHR3680 was 9.0 months (range, 0.2–40.4; Additional file 1: Table S2). A total of 165 (83.8%) patients discontinued from SHR3680 treatment, with disease progression (84/197, 42.6%) being the primary reason for treatment discontinuation. The Kaplan-Meier estimates of median TFST was 12.7 months (95% CI, 10.6–16.1).

Baseline demographics and clinical characteristics are presented in Table 1. Among the 197 patients enrolled, median age was 67.0 years (range, 45–80) and 136 (69.0%) patients had an ECOG performance status of 1. The median PSA level at baseline was 66.1 ng/mL (range, 2.7–4796.0). A total of 135 (68.5%) patients had > 5 lesions of bone metastases and 36 (18.3%) patients developed visceral metastases. As to the prior therapy, 82 (41.6%) patients had received prior chemotherapy, 50 (25.4%) had received radiotherapy, and 58 (29.4%) had a history of surgery for primary prostate cancer.

**Table 1:** Baseline demographics and clinical characteristics

	**40 mg (*N* = 3)**	**80 mg (*N*= 39)**	**160 mg (*N* = 73)**	**240 mg (*N* = 76)**	**360 mg (*N* = 3)**	**480 mg (*N* = 3)**	**Total (*N* = 197)**
Age, years							
Median	68	68	67	66	66	63	67
Range	66-77	45-80	54-80	49-79	66-79	58-71	45-80
ECOG performance status							
0	2 (66.7)	13 (33.3)	20 (27.4)	26 (34.2)	0	0	61 (31.0)
1	1 (33.3)	26 (66.7)	53 (72.6)	50 (65.8)	3 (100)	3 (100)	136 (69.0)
Gleason score							
<9	2 (66.7)	18 (46.2)	43 (58.9)	34 (44.7)	1 (33.3)	2 (66.7)	100 (50.8)
≥9	1 (33.3)	14 (35.9)	28 (38.4)	37 (48.7)	1 (33.3)	1 (33.3)	82 (41.6)
Unknown	0	7 (17.9)	2 (2.7)	5 (6.6)	1 (33.3)	0	15 (7.6)
PSA (ng/mL)							
Median	34.3	70.6	73.7	62.2	112.7	21.5	66.1
Range	9.7-36.8	2.7-2384.0	3.7-4796.0	3.3-3382.0	19.7-757.8	11.0-96.5	2.7-4796.0
Alkaline phosphatase							
Normal	3 (100)	28 (71.8)	56 (76.7)	43 (56.6)	1 (33.3)	2 (66.7)	133 (67.5)
Abnormal	0	11 (28.2)	17 (23.3)	33 (43.4)	2 (66.7)	1 (33.3)	64 (32.5)
Number of bone metastases							
≤5	1 (33.3)	12 (30.8)	23 (31.5)	24 (31.6)	1 (33.3)	1 (33.3)	62 (31.5)
>5	2 (66.7)	27 (69.2)	50 (68.5)	52 (68.4)	2 (66.7)	2 (66.7)	135 (68.5)
Visceral metastases*							
Yes	0	6 (15.4)	13 (17.8)	15 (19.7)	1 (33.3)	1 (33.3)	36 (18.3)
No	3 (100)	33 (84.6)	60 (82.2)	61 (80.3)	2 (66.7)	2 (66.7)	161 (81.7)
Prior treatment							
Surgery of primary tumor	2 (66.7)	12 (30.8)	24 (32.9)	19 (25.0)	0	1 (33.3)	58 (29.4)
Chemotherapy	0	19 (48.7)	29 (39.7)	32 (42.1)	0	2 (66.7)	82 (41.6)
Radiotherapy	1 (33.3)	12 (30.8)	18 (24.7)	18 (23.7)	0	1 (33.3)	50 (25.4)

Data are *N* (%), unless otherwise specified. * Metastasis to lymph node only are excluded. Abbreviations: *ECOG* Eastern Cooperative Oncology Group; *PSA* prostate-specific antigen

### Safety

In the 18 patients enrolled in the dose-escalation phase 1 portion, no protocol-defined DLTs were reported. Therefore, MTD was not reached.

A total of 186 (94.4%) of the 197 patients among the six dose groups experienced at least one AE of any cause. Among them, AEs in 116 (58.9%) patients were considered as treatment-related, and no dose-related trends were noted for any treatment-related adverse event (TRAE; Table 2, Additional file 1: Table S3). The most common treatment-related adverse events (TRAEs) were proteinuria (27 patients, 13.7%), hot flush (22 patients, 11.2%), and decreased white blood cell count (19 patients, 9.6%). Most TRAEs were grade 1 and grade 2. TRAEs of grade ≥ 3 occurred in only 23 (11.7%) patients, with the most common one being decreased white blood cell count (3 patients, 1.5%). There were two (1.0%) patients who had serious TRAE, including hypokalemia, pneumonia, and bone pain.

**Table 2:** Treatment-related adverse events

	**All patients (*N*** ** = 197)**	
	Any grade	Grade ≥3
Any	116 (58.9)	23 (11.7)
Proteinuria	27 (13.7)	0
Hot flush	22 (11.2)	0
White blood cell count decreased	19 (9.6)	3 (1.5)
Neutrophil count decreased	14 (7.1)	2 (1.0)
Asthenia	13 (6.6)	0
Occult blood positive	12 (6.1)	0
Aspartate aminotransferase increased	12 (6.1)	0
Bilirubin conjugated increased	11 (5.6)	0
Platelet count decreased	10 (5.1)	0
Alanine aminotransferase increased	9 (4.6)	1 (0.5)
Blood thyroid stimulating hormone increased	8 (4.1)	0
Hypertriglyceridemia	7 (3.6)	1 (0.5)
Decreased appetite	7 (3.6)	1 (0.5)
Hypertension	7 (3.6)	2 (1.0)
Gynecomastia	7 (3.6)	1 (0.5)
Anemia	7 (3.6)	1 (0.5)

Data are *N* (%). Treatment-related adverse events of any grade occurring in ≥3% of total patients are listed

Treatment was interrupted in eight (4.1%) patients due to TRAEs (Additional file 1: Table S4). Dose reduction owing to TRAE was reported in only one (0.5%) patient (grade 2, neutrophil count decreased; grade 2, white blood cell count decreased). Three (1.5%) patients discontinued treatment owing to TRAEs, including hypokalemia, bone pain, and anemia (each in one patient, 0.5%). No treatment-related deaths were reported.

### PK parameters

A total of 41 patients were included for SHR3680 concentration analysis and 38 patients for PK parameter analysis at steady state. After a single dose administration, SHR3680 was rapidly absorbed, with a median time of maximum observed plasma concentration (*T*_max_) of 2.0–18.0 h. The exposure of SHR3680 increased in a dose-dependent manner, and decreased slowly, with a geomean terminal elimination half-life (*T*_1/2_) of 76.7–89.6 h (Additional file 1: Table S5). After multiple administration, the concentration of SHR3680 reached steady state after 15 days of daily treatment. The maximum observed plasma concentration (*C*_max_) and area under the plasma concentration-time curve (AUC_0-24h_) increased in a nearly dose-proportional manner from 40 mg to 240 mg per day dose range, but the increase of exposure was slow down between the 360 mg to 480 mg per day dose range (Additional file 1: Table S6).

### Efficacy

**Fig. 2:**
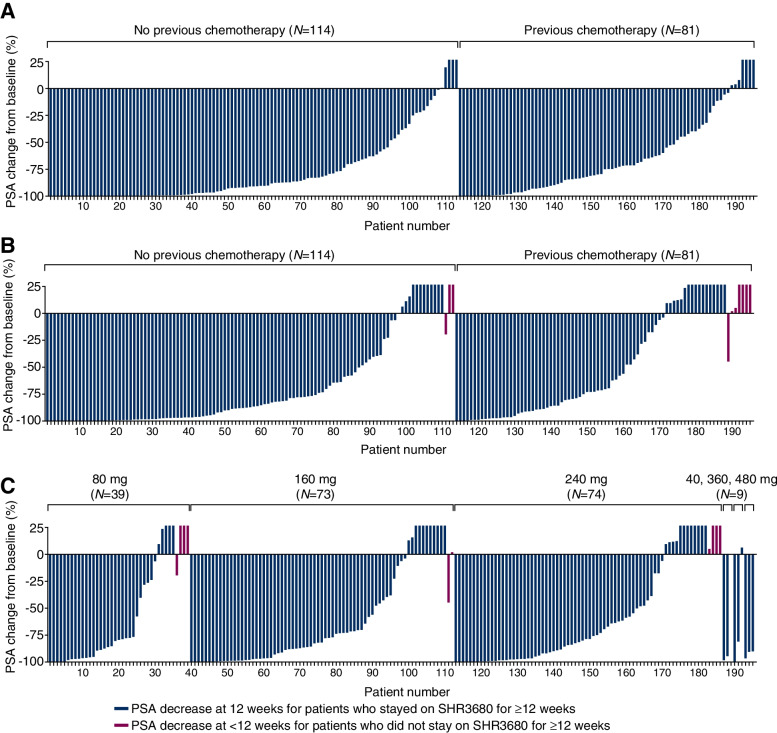
Percentage change in PSA from baseline. **A** Maximum decrease of PSA from baseline. **B** Decrease of PSA at week 12 from baseline. **C** Decrease of PSA at week 12 by dose. Two patients from 240 mg group with only baseline PSA results but no post-baseline PSA results were not included in the waterfall plots

At week 12, 134 (68.0%, 95% CI, 61.0–74.5) of the 197 patients achieved PSA response (≥ 50% decrease in PSA). The PSA responses at week 12 were noted across all dose groups, and no obvious dose-dependent activity benefits were found (Table 3). The PSA responses at week 12 in patients with and without prior chemotherapy were 57.3% (95% CI, 45.9–68.2) and 75.7% (95% CI, 66.8–83.2), respectively (Additional file 1: Table S7); other subgroup analysis results are listed in Additional file 1: Table S8. Throughout the entire treatment course, proportion of patients with a maximum PSA decrease of ≥ 50% from baseline was 78.2% (95% CI, 71.7–83.7) and of ≥ 90% was 43.7% (95% CI, 36.6–50.9) (Table 3, Additional file 1: Table S7). Waterfall plots showed the PSA decrease at week 12 and the maximum PSA decrease throughout treatment course (Fig. 2).

**Table 3:** Study endpoints related to response

	**40 mg** **(*N*** ** = 3)**	**80 mg** **(*N*** ** = 39)**	**160 mg** **(*N*** ** = 73)**	**240 mg** **(*N*** ** = 76)**	**360 mg** **(*N*** ** = 3)**	**480 mg** **(*N*** ** = 3)**	**Total** **(*N*** ** = 197)**
**PSA response**							
At week 12	2 (66.7, 9.4-99.2)	25 (64.1, 47.2-78.8)	51 (69.9, 58.0-80.1)	51 (67.1, 55.4-77.5)	2 (66.7, 9.4-99.2)	3 (100, 29.2-100)	134 (68.0, 61.0-74.5)
Maximum PSA decrease from baseline							
≥50%	3 (100, 29.2-100)	28 (71.8, 55.1-85.0)	61 (83.6, 73.0-91.2)	57 (75.0, 63.7-84.2)	2 (66.7, 9.4-99.2)	3 (100, 29.2-100)	154 (78.2, 71.7-83.7)
≥90%	2 (66.7, 9.4-99.2)	16 (41.0, 25.6-57.9)	32 (43.8, 32.2-55.9)	32 (42.1, 30.9-54.0)	1 (33.3, 0.8-90.6)	3 (100, 29.2-100)	86 (43.7, 36.6-50.9)
**Radiological response**							
**Number of evaluable patients**	1	11	19	26	3	1	61
CR	0	1 (9.1)	0	2 (7.7)	0	0	3 (4.9)
PR	0	2 (18.2)	6 (31.6)	8 (30.8)	1 (33.3)	1 (100.0)	18 (29.5)
SD	1 (100.0)	4 (36.4)	13 (68.4)	12 (46.2)	2 (66.7)	0	32 (52.5)
PD	0	3 (27.3)	0	3 (11.5)	0	0	6 (9.8)
NE	0	1 (9.1)	0	1 (3.8)	0	0	2 (3.3)
Objective response	0 (0, 0-97.5)	3 (27.3, 6.0-61.0)	6 (31.6, 12.6-56.6)	10 (38.5, 20.2-59.4)	1 (33.3, 0.8-90.6)	1 (100, 2.5-100)	21 (34.4, 22.7-47.7)
Disease control	1 (100, 2.5-100)	7 (63.6, 30.8-89.1)	19 (100, 82.4-100)	22 (84.6, 65.1-95.6)	3 (100, 29.2-100)	1 (100, 2.5-100)	53 (86.9, 75.8-94.2)
**Stable disease in bone at week 12**	3 (100, 29.2-100)	35 (89.7, 85.5-99.9)	66 (90.4, 81.2-96.1)	64 (84.2, 80.7-95.9)	3 (100, 29.2-100)	3 (100, 29.2-100)	174 (88.3, 87.2-95.5)

Data are *N* (%, 95% CI) or *N* (%). Abbreviations: *PSA* prostate-specific antigen; *CR* complete response; *PR* partial response; *SD* stable disease; *PD* progressive disease; *NE* not evaluable

As of data cutoff, PSA progression events occurred in 113 (57.4%) patients. Median time to PSA progression was 8.3 months (95% CI, 5.6–11.0) in all patients, 8.3 months (95% CI, 4.8–13.8) in patients with prior chemotherapy, and 8.3 months (95% CI, 5.5–11.0) in patients without prior chemotherapy (Additional file 1: Figure S1). The two Kaplan-Meier curves for time to PSA progression in patients with and without prior chemotherapy overlapped during the study period without clear separation.

A total of 21 (34.4%, 22.7–47.7) of the 61 patients achieved a confirmed radiological objective response, including three (4.9%) patients with CR and 18 (29.5%) patients with PR (Table 3). The ORR in patients with prior chemotherapy was 20.8% (95% CI, 7.1–42.2) and in those without prior chemotherapy was 43.2% (95% CI, 27.1–60.5; Additional file 1: Table S9). The number of patients who had a disease control was 53 (86.9%, 95% CI, 75.8–94.2). Bone scan at week 12 revealed that 174 (88.3%; 95% CI, 87.2–95.5) patients had stable disease in bone, including 69 (84.1%; 95% CI, 78.0–93.8) with prior chemotherapy and 105 (91.3%; 95% CI, 89.7–98.5) without prior chemotherapy.

**Fig. 3:**
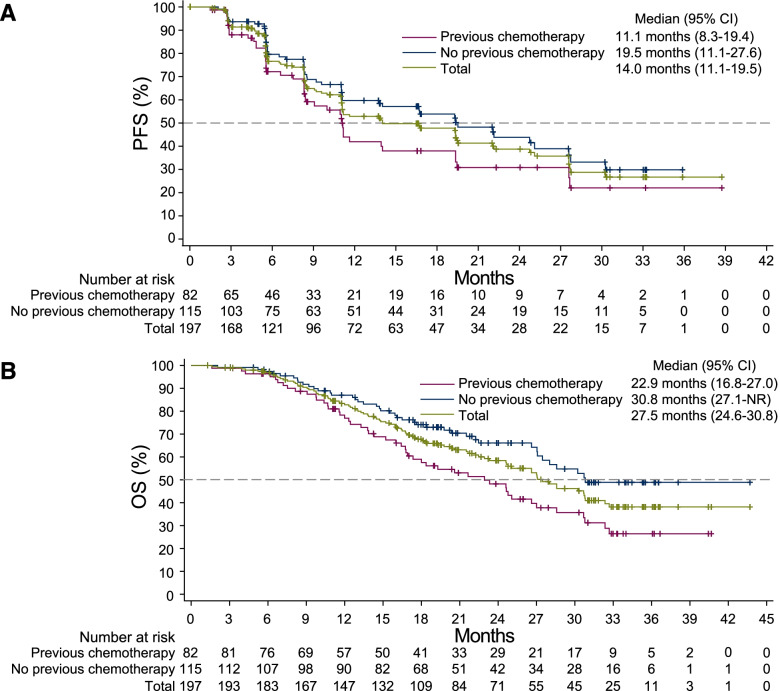
Kaplan-Meier estimates of radiological progression-free survival and overall survival. **A** Radiological progression-free survival. **B** Overall survival

Ninety-seven (49.2%) patients had PFS events (radiological progression or death) by the time of data cutoff. The median radiological PFS was 14.0 months (95% CI, 11.1–19.5; Fig. 3A). For patients with prior chemotherapy and those without prior chemotherapy, median radiological PFS was 11.1 months (95% CI, 8.3–19.4) and 19.5 months (95% CI, 11.1–27.6), respectively (Fig. 3A). As of data cutoff, 91 (46.2%) patients died. The median overall survival was 27.5 months (95% CI, 24.6–30.8; Fig. 3B). Subgroup analyses further indicated that patients with normal baseline alkaline phosphatase level, Gleason score < 9, or disease duration > 2 years had a relatively longer median PFS and OS (Additional file 1: Figure S2 and Figure S3).

## Discussion

We reported here the results of the first-in-human phase 1/2 study of a novel AR antagonist SHR3680, which has a lower distribution in the brain and decreased risk to induce seizure than enzalutamide as revealed in animal model (Additional file 1: Figure S4). The results showed that SHR3680 was well-tolerant and safe in patients with metastatic CRPC, with promising anti-tumor activity.

In the dose-escalation phase 1 portion, no DLT event occurred in any dose group ranging from 40 mg to 480 mg per day, indicating a high tolerability of SHR3680. Of note, the drug exposure of SHR3680 in 160 mg per day dose group was equivalent to that of enzalutamide in 360 mg per day dose group (*C*_max_, 23.9 μg/mL vs 25.1 μg/mL; AUC_0-τ_, 459 μg*h/mL vs 502 μg*h/mL), and the drug exposure of SHR3680 in 480 mg per day group was about twice that of SHR3680 160 mg per day group (*C*_max_, 49.7 μg/mL vs 25.1 μg/mL; AUC_0-τ_, 976 μg*h/mL vs 502 μg*h/mL) [12]. Considering that the preclinical anti-tumor activity of SHR3680 was comparable to that of enzalutamide and the MTD of enzalutamide was 240 mg per day [7], a higher dose for escalation was not considered in our study.

The safety profile of SHR3680 was favorable and similar with that of enzalutamide [7]. Only 11.7% of patients had grade ≥ 3 TRAEs, and 1.0% of patients had serious TRAEs. The incidence of TRAE was not dose-dependent. In 360 mg and 480 mg per day dose groups, despite long duration of drug exposure (mean 22.1 and 23.6 months, respectively), no new safety signals were identified.

None of the 197 patients received SHR3680 treatment in this study reported any grade of seizure. In the phase 1/2 study of enzalutamide, seizure occurred in three patients (each in 360 mg, 480 mg, and 600 mg per day group). In the phase 3 AFFIRM trial, although patients who had a risk of seizure were excluded, the incidence of seizures in the enzalutamide 160 mg per day group was still 0.9%, while no seizure occurred in placebo group [7, 8, 13]. Therefore, consistent with the results of preclinical studies (data on file, Hengrui), this study strongly supported the advantage of SHR3680 in reducing the risk of seizure compared with similar drugs.

Fatigue is another frequent and potential AE of AR antagonist. In the phase 1/2 study of enzalutamide, grade 3–4 fatigue occurred in 11% (16/140) of patients; of them, one patient discontinued enzalutamide treatment due to fatigue [7]. In our study, no grade 3–4 fatigue was reported, and no dose reduction, treatment interruption, or treatment discontinuation were induced by fatigue.

In patients without previous chemotherapy, the PSA response rate at week 12 of SHR3680 was 75.7%, which was comparable to that of apalutamide (48.0–88.0%) and darolutamide (65.5–83.3%) in studies which had similar inclusion criteria and baseline clinical characteristics as our study [14–18]. In patients with a history of chemotherapy, the PSA response rate at week 12 was 57.3% and was numerically higher than that of darolutamide (32.3%) [14]. Even though PSA decrease could be just an indicator of the mechanism of action of SHR3680 as an AR antagonist, the radiographic assessment results obtained from both chemotherapy-naïve (ORR 43.2%; median radiographic PFS 19.5 months) and post-chemotherapy patient populations (ORR 20.8%; median radiographic PFS 11.1 months), which were also comparable to those of other second-generation of AR antagonists, provided further evidence for the anti-tumor activity of SHR3680 [14–18].

Among the three expanded dose groups, the plasma SHR3680 concentrations in 160 mg and 240 mg groups were higher than that in the 80 mg group, but the improvement of PSA response in the 160 mg and 240 mg groups was marginal when compared with that in the 80 mg group, suggesting the effect of SHR3680 in reducing PSA level may have reached saturation at a dose of 160–240 mg daily. Such phenomenon of saturation in PSA reduction was also observed in cases of enzalutamide and darolutamide [7, 14]. In contrast, we found that the response rate of soft tissue lesions at SHR3680 240 mg per day was higher than that at low doses.

## Conclusions

SHR3680 was well tolerated and safe in patients with metastatic CRPC. It had encouraging efficacy in PSA reduction and anti-tumor activity in patients with or without prior chemotherapy. The results of this study supported further investigation of SHR3680, at a planned dose of 240 mg per day, in a randomized controlled phase 3 trial (NCT03520478) in patients with metastatic hormone sensitive prostate cancer.

## Supplementary Information

**Additional file 1: Figure S1.** Kaplan-Meier estimates of time to PSA progression. **Figure S2.** Kaplan-Meier estimates of radiological PFS in subgroups. **Figure S3.** Kaplan-Meier estimates of OS in subgroups. **Figure S4.** Preclinical data of SHR3680 in vivo. **Table S1.** Lists of study sites and investigators. **Table S2.** Drug exposure. **Table S3.** Treatment-related adverse events in each dose group. **Table S4.** Dose reduction, treatment interruption and discontinuation due to TRAEs. **Table S5.** Pharmacokinetic parameters for SHR3680 after single-dose. **Table S6.** Pharmacokinetic parameters for SHR3680 at steady state. **Table S7.** PSA decline in patients with or without prior chemotherapy. **Table S8.** PSA response at week 12 in subgroups. **Table S9.** Radiological response in patients with or without prior chemotherapy.

## Data Availability

The datasets used and/or analyzed during the current study are available from the corresponding author on reasonable request.

## Abbreviations

AE: Adverse event
AR: Androgen receptor
CR: Complete response
CRPC: Castration-resistant prostate cancer
DCR: Disease control rate
DLT: Dose-limiting toxicity
ECOG: Eastern Cooperative Oncology Group
GABAa: γ-Aminobutyric acid
MTD: Maximum tolerable dose
ORR: Objective response rate
OS: Overall survival
PCWG2: Prostate Cancer Working Group
PFS: Progression-free survival
PK: Pharmacokinetics
PR: Partial response
PSA: Prostate-specific antigen
RECIST: Response Evaluation Criteria in Solid Tumors
TFST: Time to first subsequent therapy
TRAE: Treatment-related adverse event
