# Supplementary material for: Activity and safety of SHR3680, a novel antiandrogen, in patients with metastatic castration-resistant prostate cancer: a phase I/II trial
Source: BMC Med. 2022 Mar 4;20:84. doi: 10.1186/s12916-022-02263-x (PMC8895828; doi:10.1186/s12916-022-02263-x)
Supplement: Supplementary file 1 — Additional file 1: Figure S1. Kaplan-Meier estimates of time to PSA progression. Figure S2. Kaplan-Meier estimates of radiological PFS in subgroups. Figure S3. Kaplan-Meier estimates of OS in subgroups. Figure S4. Preclinical data of SHR3680 in vivo. Table S1. Lists of study sites and investigators. Table S2. Drug exposure. Table S3. Treatment-related adverse events in each dose group. Table S4. Dose reduction, treatment interruption and discontinuation due to TRAEs. Table S5. Pharmacokinetic parameters for SHR3680 after single-dose. Table S6. Pharmacokinetic parameters for SHR3680 at steady state. Table S7. PSA decline in patients with or without prior chemotherapy. Table S8. PSA response at week 12 in subgroups. Table S9. Radiological response in patients with or without prior chemotherapy. [file 12916_2022_2263_MOESM1_ESM.docx]

**Supplementary**

**Supplement to:** Xiaojian Qin, et al. Activity and safety of SHR3680, a novel antiandrogen, in patients with metastatic castration-resistant prostate cancer: A phase I/II trial

**Table of contents**

[Figure S1. Kaplan-Meier estimates of time to PSA progression. 2](#_Toc90644752)

[Figure S2. Kaplan-Meier estimates of radiological PFS in subgroups. 3](#_Toc90644753)

[Figure S3. Kaplan-Meier estimates of OS in subgroups. 5](#_Toc90644754)

[Figure S4. Preclinical data of SHR3680 *in vivo*. 6](#_Toc90644755)

[Table S1. Lists of study sites and investigators. 7](#_Toc90644756)

[Table S2. Drug exposure. 8](#_Toc90644757)

[Table S3. Treatment-related adverse events in each dose group. 9](#_Toc90644758)

[Table S4. Dose reduction, treatment interruption and discontinuation due to TRAEs. 12](#_Toc90644759)

[Table S5. Pharmacokinetic parameters for SHR3680 after single-dose. 14](#_Toc90644760)

[Table S6. Pharmacokinetic parameters for SHR3680 at steady state. 15](#_Toc90644761)

[Table S7. PSA decline in patients with or without prior chemotherapy. 16](#_Toc90644762)

[Table S8. PSA response at week 12 in subgroups. 17](#_Toc90644763)

[Table S9. Radiological response in patients with or without prior chemotherapy. 18](#_Toc90644764)

# Figure S1. Kaplan-Meier estimates of time to PSA progression.


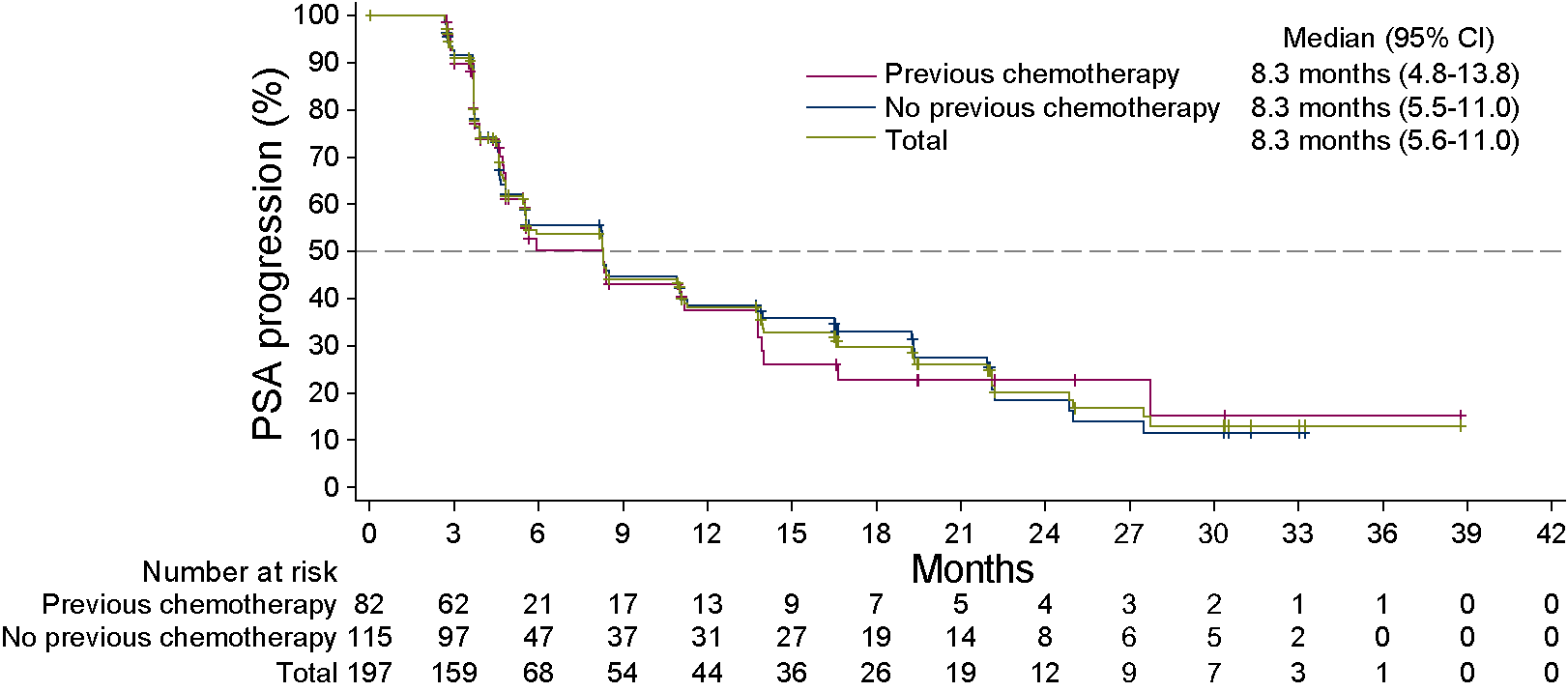


# Figure S2. Kaplan-Meier estimates of radiological PFS in subgroups.


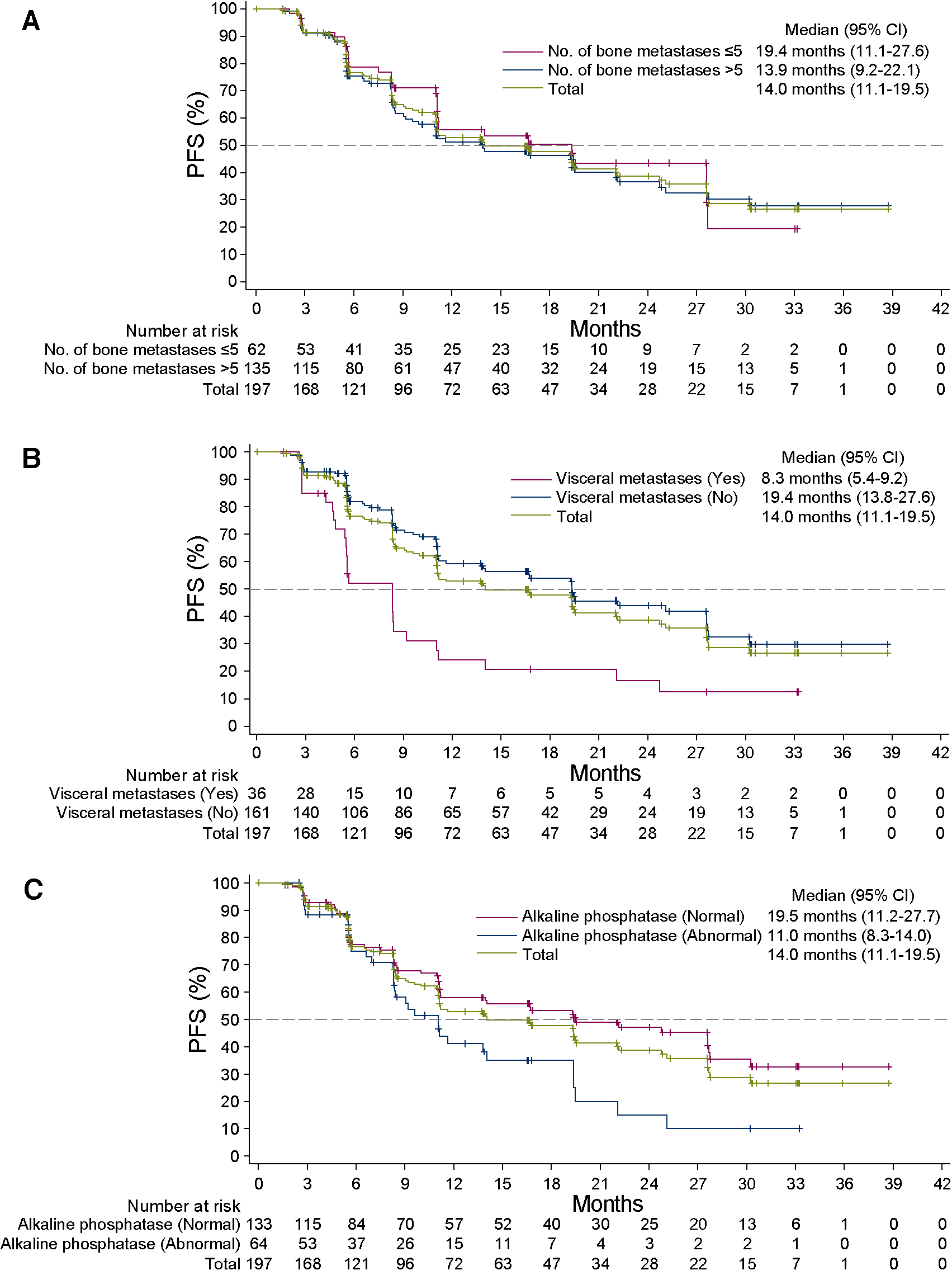


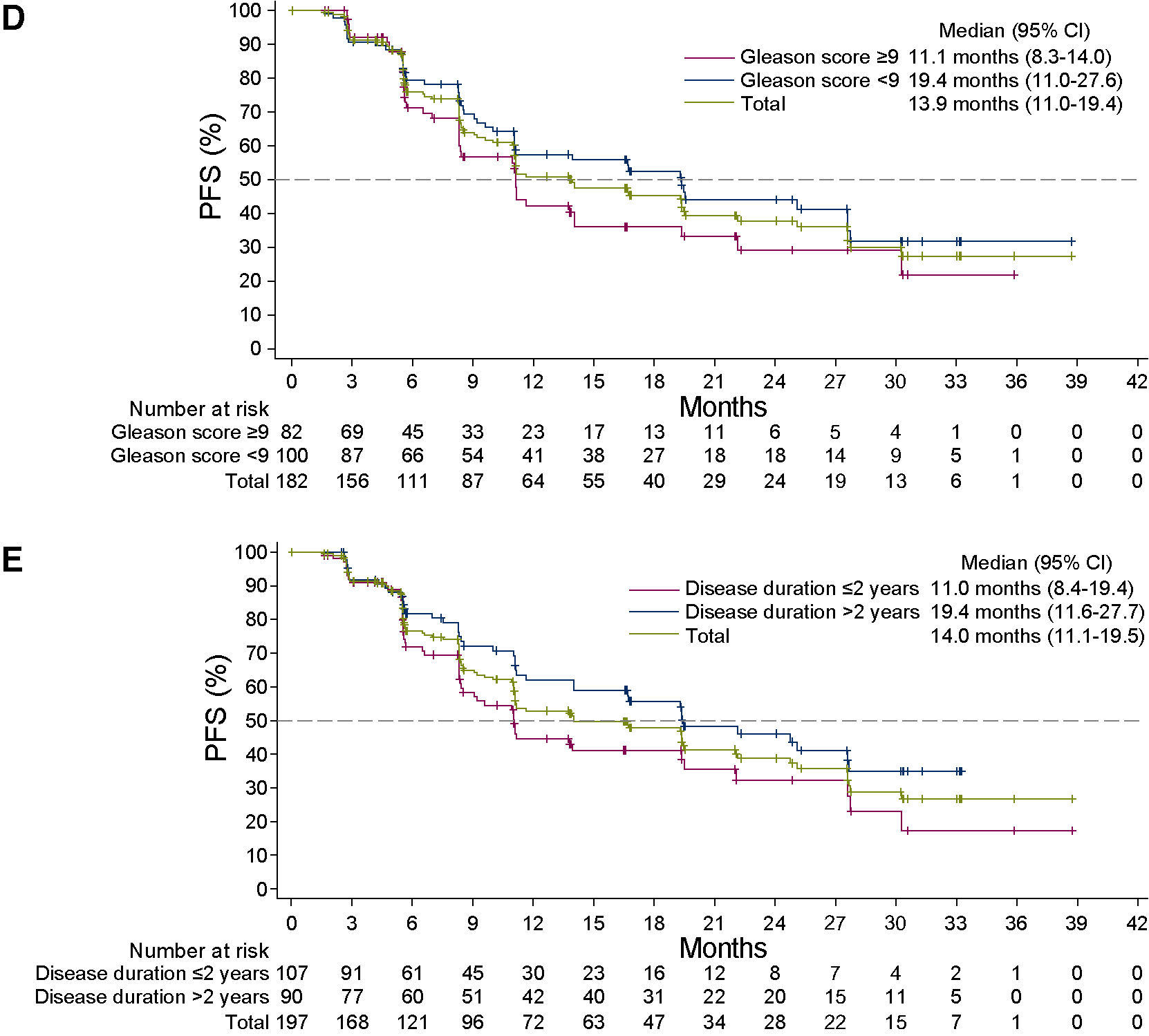


# Figure S3. Kaplan-Meier estimates of OS in subgroups.


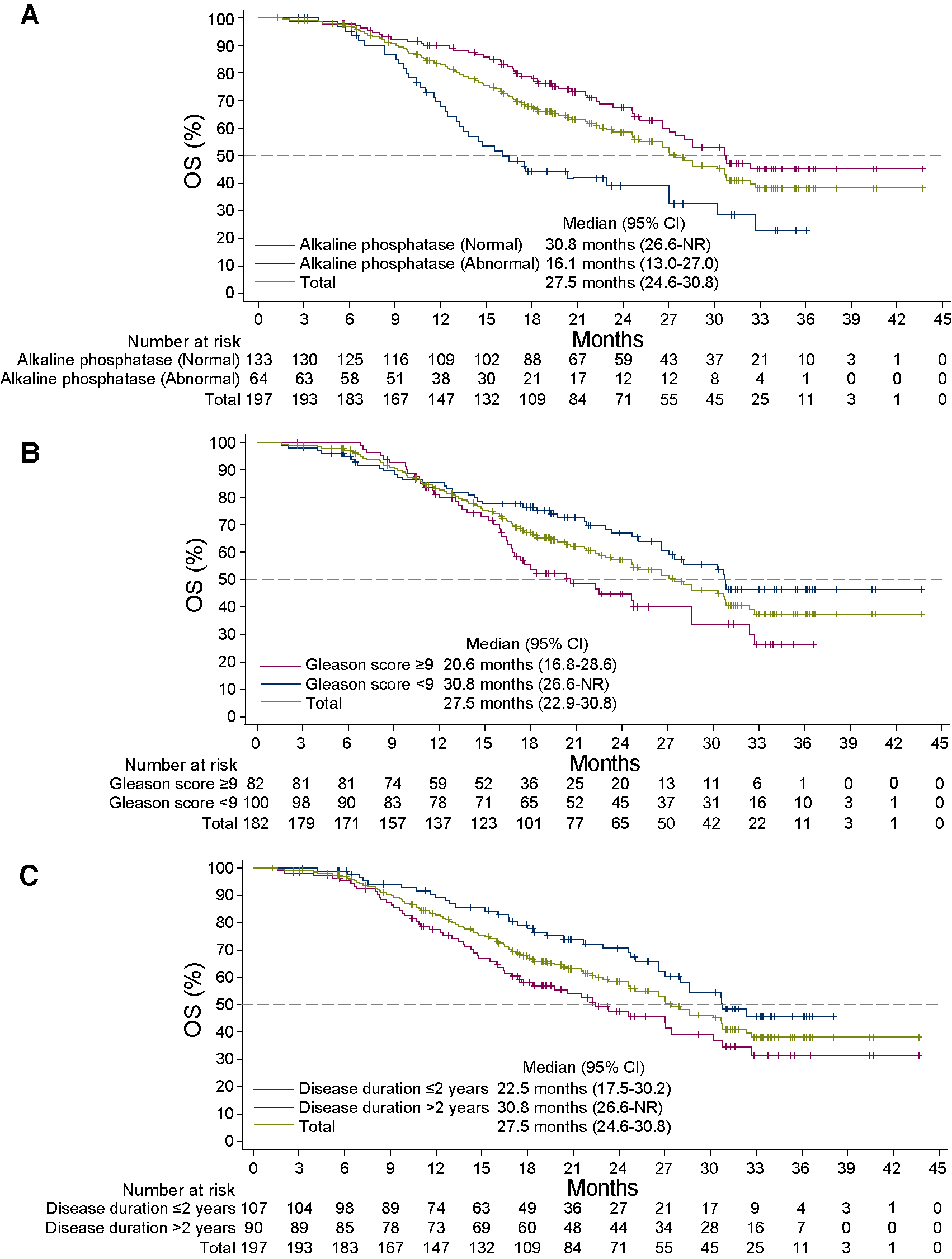


# Figure S4. Preclinical data of SHR3680 *in vivo*.

The preclinical study showed SHR3680 has a lower distribution in the brain and decreased risk of seizure than enzalutamide.

(A) The ratio of brain to plasma concentration with SHR3680 was lower than that of enzalutamide. Concentrations of SHR3680 and enzalutamide in plasma and brain of C57BL/6J mice 1 hour and 4 hours after intragastric administration were determined by liquid chromatography tandem mass spectrometry (LC/MS/MS). All mice were male with a body weight of 19.45±0.19 g. The height and error bar of each column represents mean±standard deviation (SD) of measurements from three independent mice.

(B) Administration of SHR3680 led to prolonged latency durations to myoclonic seizure and straub’s tail response than enzalutamide. C57BL/6J mice (male, weight 19.45±0.19 g) received a single oral dose of SHR3680 or enzalutamide, and the latency time to myoclonic seizure and straub’s tail response of mice were recorded within 60 minutes. All data were expressed as mean±SD. One asterisk indicates P < 0.05 compared with enzalutamide group, two asterisks, P < 0.01; and three asterisks, P < 0.001. Ten mice were used in each group.

(C) Administration of SHR3680 led to lower incidences of myoclonic seizure and straub’s tail response than enzalutamide. Ten mice were used in each group.


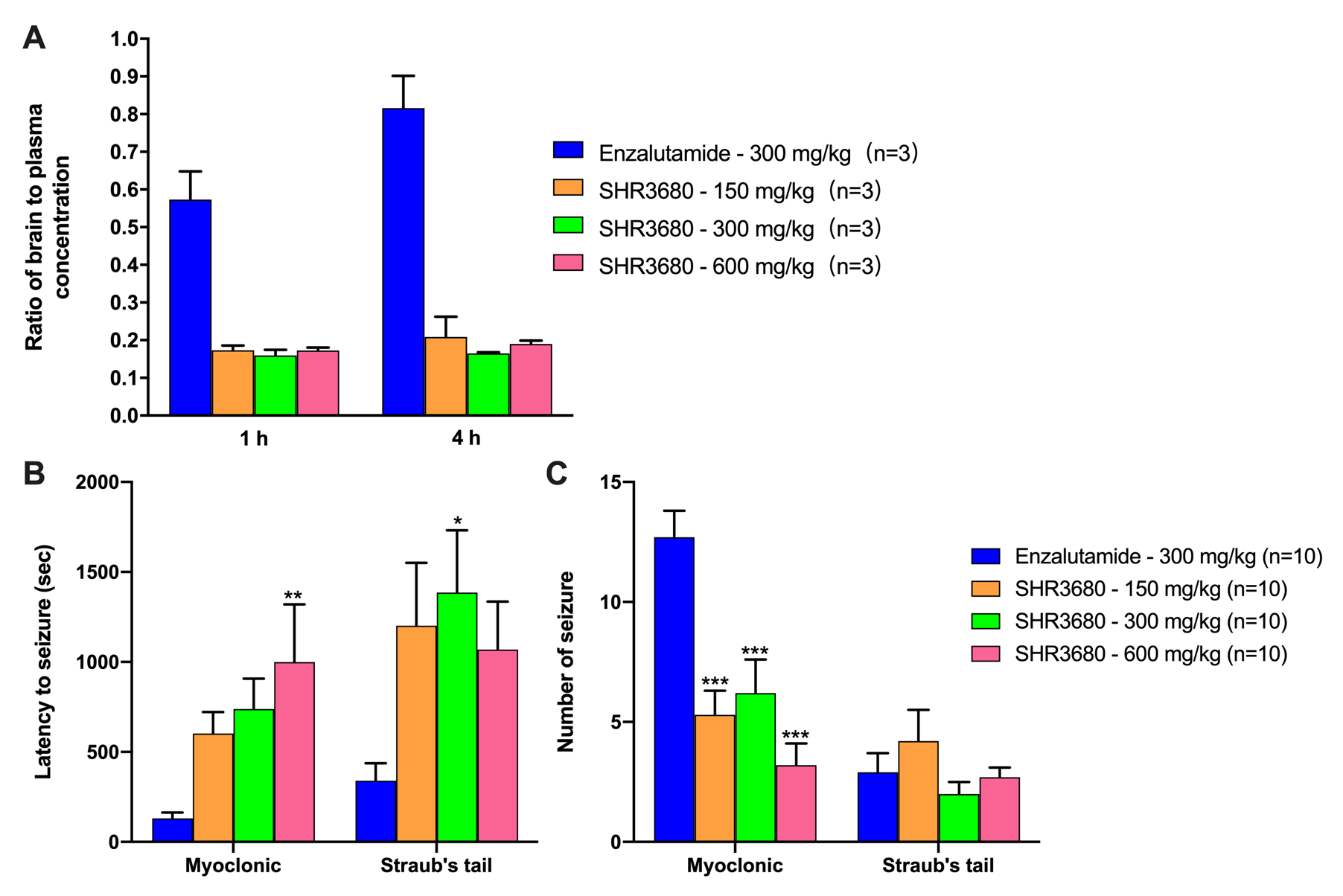


# Table S1. Lists of study sites and investigators.

| **Investigators** | **Study sites** | **Number of patients enrolled** |
| --- | --- | --- |
| Dingwe Ye / Xiaojian Qin / Weijie Gu / Dongmei Ji | Fudan University Shanghai Cancer Center, Shanghai, China | 42 |
| Weiqing Han | Hunan Cancer Hospital, Changsha, China | 34 |
| Hong Luo | Chongqing Cancer Hospital, Chongqing, China | 20 |
| Chuanjun Du | The Second Affiliated hospital of Zhejiang University School of Medicine, Hangzhou, China | 18 |
| Qing Zou | Jiangsu Cancer Hospital, Jiangsu, China | 17 |
| Zhongquan Sun | Huadong Hospital Affiliated to Fudan University, Shanghai, China | 16 |
| Chaohong He | Cancer Hospital of Henan Province, Zhengzhou, China | 14 |
| Shaoxing Zhu | Zhejiang Cancer Hospital, Hangzhou, China | 12 |
| Tie Chong | The Second Affiliated Hospital of Xi’an Jiaotong University, Xi'an, China | 10 |
| Xin Yao | Tianjin Cancer Hospital, Tianjin, China | 8 |
| Ben Wan | Beijing Hospital, Beijing, China | 6 |

# Table S2. Drug exposure.

|  | **40 mg**  **(*N* = 3)** | **80 mg**  **(*N* = 39)** | **160 mg**  **(*N* = 73)** | **240 mg**  **(*N* = 76)** | **360 mg**  **(*N* = 3)** | **480 mg**  **(*N* = 3)** | **Total**  **(*N* = 197)** |
| --- | --- | --- | --- | --- | --- | --- | --- |
| Duration of drug exposure, months, median (range) | 17.7 (4.4-32.9) | 11.4 (0.7-40.4) | 10.1 (1.6-36.5) | 8.3 (0.2-33.9) | 25.1 (5.7-35.4) | 19.5 (18.4-32.9) | 9.0 (0.2-40.4) |

# Table S3. Treatment-related adverse events in each dose group.

|  | **40 mg**  **(*N* = 3)** | | **80 mg**  **(*N* = 39)** | | **160 mg**  **(*N* = 73)** | | **240 mg**  **(*N* = 76)** | | **360 mg**  **(*N* = 3)** | | **480 mg**  **(*N* = 3)** | | **Total**  **(*N* = 197)** | |
| --- | --- | --- | --- | --- | --- | --- | --- | --- | --- | --- | --- | --- | --- | --- |
|  | Any grade | Grade ≥3 | Any grade | Grade ≥3 | Any grade | Grade ≥3 | Any grade | Grade ≥3 | Any grade | Grade ≥3 | Any grade | Grade ≥3 | Any grade | Grade ≥3 |
| Any | 3 (100.0) | 2 (66.7) | 19 (48.7) | 4 (10.3) | 43 (58.9) | 3 (4.1) | 45 (59.2) | 13 (17.1) | 3 (100.0) | 1 (33.3) | 3 (100.0) | 0 | 116 (58.9) | 23 (11.7) |
| Proteinuria | 1 (33.3) | 0 | 5 (12.8) | 0 | 7 (9.6) | 0 | 11 (14.5) | 0 | 1 (33.3) | 0 | 2 (66.7) | 0 | 27 (13.7) | 0 |
| Hot flush | 3 (100.0) | 0 | 4 (10.3) | 0 | 6 (8.2) | 0 | 6 (7.9) | 0 | 1 (33.3) | 0 | 2 (66.7) | 0 | 22 (11.2) | 0 |
| White blood cell count decreased | 0 | 0 | 6 (15.4) | 1 (2.6) | 5 (6.8) | 0 | 8 (10.5) | 2 (2.6) | 0 | 0 | 0 | 0 | 19 (9.6) | 3 (1.5) |
| Neutrophil count decreased | 0 | 0 | 5 (12.8) | 1 (2.6) | 4 (5.5) | 0 | 4 (5.3) | 1 (1.3) | 0 | 0 | 1 (33.3) | 0 | 14 (7.1) | 2 (1.0) |
| Asthenia | 1 (33.3) | 0 | 2 (5.1) | 0 | 3 (4.1) | 0 | 5 (6.6) | 0 | 2 (66.7) | 0 | 0 | 0 | 13 (6.6) | 0 |
| Occult blood positive | 0 | 0 | 3 (7.7) | 0 | 3 (4.1) | 0 | 6 (7.9) | 0 | 0 | 0 | 0 | 0 | 12 (6.1) | 0 |
| Aspartate aminotransferase increased | 2 (66.7) | 0 | 4 (10.3) | 0 | 2 (2.7) | 0 | 2 (2.6) | 0 | 1 (33.3) | 0 | 1 (33.3) | 0 | 12 (6.1) | 0 |
| Bilirubin conjugated increased | 2 (66.7) | 0 | 6 (15.4) | 0 | 1 (1.4) | 0 | 2 (2.6) | 0 | 0 | 0 | 0 | 0 | 11 (5.6) | 0 |
| Platelet count decreased | 0 | 0 | 2 (5.1) | 0 | 1 (1.4) | 0 | 7 (9.2) | 0 | 0 | 0 | 0 | 0 | 10 (5.1) | 0 |
| Alanine aminotransferase increased | 1 (33.3) | 0 | 3 (7.7) | 0 | 1 (1.4) | 0 | 3 (3.9) | 1 (1.3) | 0 | 0 | 1 (33.3) | 0 | 9 (4.6) | 1 (0.5) |
| Blood thyroid stimulating hormone increased | 0 | 0 | 0 | 0 | 4 (5.5) | 0 | 2 (2.6) | 0 | 1 (33.3) | 0 | 1 (33.3) | 0 | 8 (4.1) | 0 |
| Hypertriglyceridemia | 0 | 0 | 1 (2.6) | 1 (2.6) | 5 (6.8) | 0 | 1 (1.3) | 0 | 0 | 0 | 0 | 0 | 7 (3.6) | 1 (0.5) |
| Decreased appetite | 0 | 0 | 4 (10.3) | 0 | 0 | 0 | 3 (3.9) | 1 (1.3) | 0 | 0 | 0 | 0 | 7 (3.6) | 1 (0.5) |
| Hypertension | 1 (33.3) | 1 (33.3) | 1 (2.6) | 1 (2.6) | 4 (5.5) | 0 | 0 | 0 | 1 (33.3) | 0 | 0 | 0 | 7 (3.6) | 2 (1.0) |
| Gynecomastia | 2 (66.7) | 0 | 4 (10.3) | 1 (2.6) | 1 (1.4) | 0 | 0 | 0 | 0 | 0 | 0 | 0 | 7 (3.6) | 1 (0.5) |
| Anemia | 0 | 0 | 2 (5.1) | 0 | 1 (1.4) | 0 | 4 (5.3) | 1 (1.3) | 0 | 0 | 0 | 0 | 7 (3.6) | 1 (0.5) |

Data are *N* (%). Treatment-related adverse events of any grade occurring in ≥3% of total patients are listed.

# Table S4. Dose reduction, treatment interruption and discontinuation due to TRAEs.

|  | **40 mg**  **(*N* = 3)** | **80 mg**  **(*N* = 39)** | **160 mg**  **(*N* = 73)** | **240 mg**  **(*N* = 76)** | **360 mg**  **(*N* = 3)** | **480 mg**  **(*N* = 3)** | **Total**  **(*N* = 197)** |
| --- | --- | --- | --- | --- | --- | --- | --- |
| **Treatment interruption or dose reduction** | **1 (33.3)** | **1 (2.6)** | **0** | **4 (5.3)** | **1 (33.3)** | **1 (33.3)** | **8 (4.1)** |
| Lipase increased | 0 | 0 | 0 | 1 (1.3) | 1 (33.3) | 0 | 2 (1.0) |
| White blood cell count decreased | 0 | 0 | 0 | 1 (1.3)* | 0 | 0 | 1 (0.5) |
| Alanine aminotransferase increased | 0 | 0 | 0 | 1 (1.3) | 0 | 0 | 1 (0.5) |
| Aspartate aminotransferase increased | 0 | 0 | 0 | 1 (1.3) | 0 | 0 | 1 (0.5) |
| Neutrophil count decreased | 0 | 0 | 0 | 1 (1.3)* | 0 | 0 | 1 (0.5) |
| Hypokalemia | 0 | 1 (2.6) | 0 | 0 | 0 | 0 | 1 (0.5) |
| Decreased appetite | 0 | 0 | 0 | 1 (1.3) | 0 | 0 | 1 (0.5) |
| Pneumonitis | 0 | 0 | 0 | 0 | 0 | 1 (33.3) | 1 (0.5) |
| Hypertension | 1 (33.3) | 0 | 0 | 0 | 0 | 0 | 1 (0.5) |
| **Treatment discontinuation** | **0** | **1 (2.6)** | **0** | **2 (2.6)** | **0** | **0** | **3 (1.5)** |
| Hypokalemia | 0 | 1 (2.6) | 0 | 0 | 0 | 0 | 1 (0.5) |
| Bone pain | 0 | 0 | 0 | 1 (1.3) | 0 | 0 | 1 (0.5) |
| Anemia | 0 | 0 | 0 | 1 (1.3) | 0 | 0 | 1 (0.5) |

Data are *N* (%). * Only one patient at the group of 240 mg had dose reduction due to treatment related decreased white blood cell count and decreased neutrophil count.

# Table S5. Pharmacokinetic parameters for SHR3680 after single-dose.

| **Dose** |  | **C_max_ (ng/mL)** | **T_max_ (h)** | **AUC_0-24h_ (h*ng/mL)** | **AUC_last_ (h*ng/mL)** | **T_1/2_ (h)** |
| --- | --- | --- | --- | --- | --- | --- |
| 40 mg (*N* = 3) | Geomean | 1440 | 2.0 | 26000 | 117000 | 78.9 |
|  | CV% | 15.9 | 0.5-8.0 | 17.5 | 7.2 | 11.7 |
| 80 mg (*N* = 10) | Geomean | 2750 | 4.0 | 55300 | 247000 | 80.9 |
|  | CV% | 17.0 | 2.0-23.6 | 20.2 | 18.1 | 20.4 |
| 160 mg (*N* = 9) | Geomean | 5540 | 6.0 | 111000 | 475000 | 76.7 |
|  | CV% | 20.1 | 2.0-11.9 | 15.6 | 17 | 27.4 |
| 240 mg (*N* = 10) | Geomean | 7620 | 7.0 | 158000 | 690000 | 89.6 |
|  | CV% | 21.2 | 2.0-24.2 | 24.1 | 27.1 | 25.8 |
| 360 mg (*N* = 3) | Geomean | 9480 | 18.0 | 165000 | 974000 | 80.3 |
|  | CV% | 18.4 | 8.0-35.6 | 11.1 | 9.7 | 34.0 |
| 480 mg (*N* = 3) | Geomean | 12800 | 8.0 | 270000 | 1280000 | 82.0 |
|  | CV% | 9.8 | 8.0-11.9 | 3.3 | 9.8 | 14.3 |

T_max_ is presented in median (range), other data are presented in mean. Abbreviation: CV, coefficient of variation; C_max_, maximum concentration; T_max_, time of maximum observed plasma concentration; AUC_0-24h_, area under plasma concentration-time curve over 0 to 24 hours; AUC_last_, area under plasma concentration-time curve over 0 to 168 hours; T_1/2_, half-life.

# Table S6. Pharmacokinetic parameters for SHR3680 at steady state.

| **Dose** |  | **C_max_ (ng/mL)** | **T_max_ (h)** | **AUC_0-24h_ (h*ng/mL)** | **T_1/2_ (h)** |
| --- | --- | --- | --- | --- | --- |
| 40 mg (*N* = 3) | Geomean | 5760 | 2.0 | 115000 | 79.8 |
|  | CV% | 9.8 | 2.0-3.9 | 5.9 | 97.7 |
| 80 mg (*N* = 10) | Geomean | 13700 | 3.0 | 263000 | 78.0 |
|  | CV% | 18.9 | 0.5-8.3 | 11.7 | 29.3 |
| 160 mg (*N* =9) | Geomean | 23900 | 2.0 | 459000 | 91.7 |
|  | CV% | 18.7 | 1.0-4.0 | 12.2 | 73.0 |
| 240 mg (*N* = 10) | Geomean | 32500 | 2.0 | 649000 | 74.9 |
|  | CV% | 22.4 | 0.5-23.6 | 30.0 | 55.9 |
| 360 mg (*N* = 3) | Geomean | 40500 | 2.0 | 783000 | 69.3 |
|  | CV% | 18.9 | 2.0-2.0 | 19.0 | 33.2 |
| 480 mg (*N* = 3) | Geomean | 49700 | 2.0 | 976000 | 66.6 |
|  | CV% | 11.8 | 0.0-8.0 | 14.4 | 25.0 |

T_max_ is presented in median (range), other data are presented in mean. Abbreviation: CV, coefficient of variation; C_max_, maximum concentration; T_max_, time of maximum observed plasma; AUC_0-24h_, area under plasma concentration-time curve over 0 to 24 hours; T_1/2_, half-life.

# Table S7. PSA decline in patients with or without prior chemotherapy.

|  | **With prior chemotherapy**  **(*N* = 82)** | **Without prior chemotherapy**  **(*N* = 115)** | **Total**  **(*N* = 197)** |
| --- | --- | --- | --- |
| PSA response at week 12 | 47 (57.3, 45.9-68.2) | 87 (75.7, 66.8-83.2) | 134 (68.0, 61.0-74.5) |
| Maximum PSA decrease from baseline |  |  |  |
| ≥50% | 60 (73.2, 62.2-82.4) | 94 (81.7, 73.5-88.3) | 154 (78.2, 71.7-83.7) |
| ≥90% | 26 (31.7, 21.9-42.9) | 60 (52.2, 42.7-61.6) | 86 (43.7, 36.6-50.9) |

Data are *N* (%, 95% CI).

# Table S8. PSA response at week 12 in subgroups.

|  | **Number of patients** | **PSA response at week 12** |
| --- | --- | --- |
| Number of bone metastases |  |  |
| ≤5 | 62 | 37 (59.7, 46.4-71.9) |
| >5 | 135 | 97 (71.9, 63.5-79.2) |
| Visceral metastases* |  |  |
| Yes | 36 | 22 (61.1, 43.5-76.9) |
| No | 161 | 112 (69.6, 61.8-76.6) |

Data are *N* (%, 95% CI). *Metastasis to lymph node only are excluded.

# Table S9. Radiological response in patients with or without prior chemotherapy.

|  | **With prior chemotherapy**  **(*N* = 82)** | **Without prior chemotherapy**  **(*N* = 115)** |
| --- | --- | --- |
| Number of patients with measurable target lesions at baseline | 24 | 37 |
| CR | 0 | 3 (8.1) |
| PR | 5 (20.8) | 13 (35.1) |
| SD | 15 (62.5) | 17 (45.9) |
| PD | 3 (12.5) | 3 (8.1) |
| NE | 1 (4.2) | 1 (2.7) |
| Objective response* | 5 (20.8, 7.1-42.2) | 16 (43.2, 27.1-60.5) |
| Disease control** | 20 (83.3, 62.6-95.3) | 33 (89.2, 74.6-97.0) |

Data are *N* (%) or *N* (%, 95% CI). * Objective response rate was number of patients with the best response of CR or PR divided by the total number of patients with measurable target lesions at baseline. ** Disease control rate was number of patients with the best response of CR, PR, or SD divided by the total number of patients with measurable target lesions at baseline.
